# Quercetin alleviates chronic renal failure by targeting the PI3k/Akt pathway

**DOI:** 10.1080/21655979.2021.1973877

**Published:** 2021-09-16

**Authors:** Haitao Tu, Duanhua Ma, Yuanyuan Luo, Shuifu Tang, Ying Li, Gangyi Chen, Liangliang Wang, Zhengkun Hou, Chuangpeng Shen, Huan Lu, Xun Zhuang, Liangyou Zhang

**Affiliations:** a Division of Nephrology, The First Affiliated Hospital of Guangzhou University of Chinese Medicine, Guangdong, Guangzhou, China; b Science and Technology Innovation Center, Guangzhou University of Chinese Medicine, Guangdong, Guangzhou, China; c Department of Intensive Care Unit, The First Affiliated Hospital of Guangzhou University of Chinese Medicine, Guangzhou, Guangdong, China; d Division of Nephrology, Chongqing Hospital of Traditional Chinese Medicine, Chongqing, China; e Department of Gastroenterology, The First Affiliated Hospital of Guangzhou University of Chinese Medicine, Guangzhou, Guangdong, China; f Department of Endocrinology, The First Affiliated Hospital of Guangzhou University of Chinese Medicine, Guangzhou, Guangdong, China; g Department of Rehabilitation Center, The First Affiliated Hospital of Guangzhou University of Chinese Medicine, Guangzhou, Guangdong, China

**Keywords:** CRF, quercetin, pik3r1, pi3k/akt

## Abstract

Chronic renal failure (CRF) threatens human health greatly and attracts worldwide concerns of health professionals in the public health sector. In our preliminary study, we found that Compound capsule (Shengqing Jiangzhuo Capsule, SQJZJN) had a significant therapeutic effect on CRF. Quercetin is one of the main components of this Compound capsule. In this study, we investigated the effect of Quercetin monomer on CRF and the regulation of PI3k/Akt pathway. Network pharmacology analysis methods were employed to analyze the SQJZJN/Quercetin/PIK3R1 network relationships. In this study, a CRF rat model was prepared using the gavage adenine solution method and detected the indicators of Creatinine (Cr), Blood Urea Nitrogen (BUN), and Uric Acid (UA). After treating the rat model with Quercetin and PIK3R1-interfering lentivirus, respectively, we observed the changes on the histological morphology of the kidney and detected apoptosis using TUNEL staining. Gene and protein expression associated with renal function were detected using qPCR, WB and immunofluorescence. Quercetin was identified as the main ingredient of SQJZJN by the network pharmacological screening and Quercetin at 1.5 and 3 g/(kg.d) concentrations could effectively alleviate the CRF symptoms, reduce the levels of Cr, BUN, and UA, and markedly inhibit cell apoptosis demonstrated by the intragastric administration. Furthermore, the protein expression of p-PI3K, p-AKT, NLRP3, caspase1, AQP1, and AQP2 in all groups was detected by immunofluorescence and western blot assays, indicating that Quercetin could reduce the expression of NLRP3, caspase1, p-PI3k, and p-Akt, and increase the expression of AQP1 and AQP2 in the renal tissues of CRF rats. Being labeled with biotin and incubated with the total protein extracted from kidney tissues, Quercetin could bind to PIK3R1. Following the PIK3R1 interference lentivirus was injected into the CRF model rats by tail vein, the CRF symptoms were effectively alleviated in the PIK3R1 interference group, consistent with the effect of Quercetin. Taken together, Quercetin, a major component of SQJZJN, might minimize renal fibrosis and apoptosis in CRF rats by inhibiting the PI3k/Akt pathway through targeting PIK3R1. By regulating AQP1 and AQP2, both water retention and toxin accumulation were reduced.

## Graphical abstract

**Figure:**
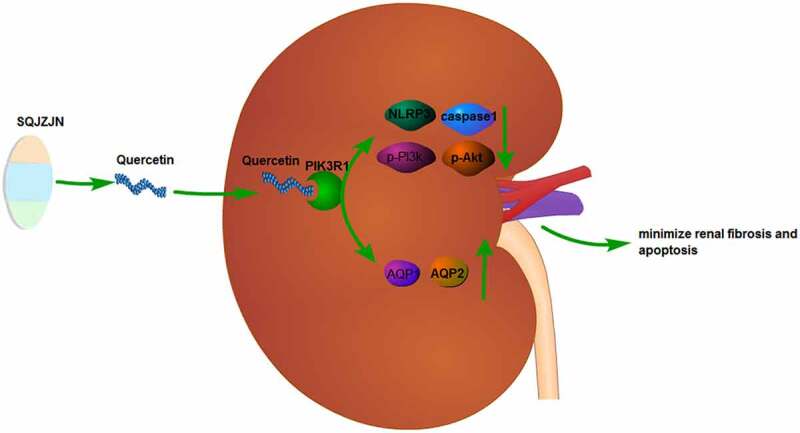

## 1 Introduction

As the economy booms, the society advances, the lifestyle of people changes, and the aging process of the population structure accelerates, tremendous changes occur in the spectrum of human disease. Chronic noninfectious diseases have evolved into global public health issues, and chronic kidney disease (CKD) is one of them^[1]^. Once CKD develops into CRF, the patient will live a reduced quality of life and it endangers his or her life and well-being. According to a survey, CRF and its end-stage renal disease have a high incidence with poor prognosis but high medical expenses. CRF is also considered as an independent risk factor of cardiovascular events, intimately associated with cardiovascular events, and cardiovascular mortality, and all-cause mortality^[2]^. The number of end-stage renal disease patients requiring renal replacement is growing rapidly. This disease not only affects people’s health greatly but also brings about a heavy financial burden on global health care^[3]^.

SQJZJN is an effective traditional Chinese medicine (TCM) prescription for CRF pathogenesis^[4]^. SQJZJN was prepared with Polygonum cuspidatum Siebold & Zucc (Chinese name, Huzhang), (Chinese name, Tufuling), the dried flower buds of (Chinese name, Huaihua), (Chinese name, Huangqi) at 1:1:1:0.6 rate. The formula is characterized by strengthening healthy qi (a concept of Chinese medicine, which would make your spleen and stomach stronger and to invigorate the Yang qi in your body), removing turbidity and detoxification, and breaking blood stasis thereby promoting tissue regeneration. This experienced prescription was developed by targeting the TCM pathogenesis of CRF, the characteristics of both the spleen and kidney failure causing turbidity and obstruction of triple energizer, integrating modern medical research findings, syndrome differentiation, and disease differentiation. SQJZJN has demonstrated a notable effect in alleviating or postponing renal fibrosis on CRF in clinical trials, animal experiments, and experiments in vitro^[4]^.

Quercetin is a polyhydroxyflavonoid. Studies have shown that Quercetin protects the kidney to some extent by lowering glucose, reducing oxidative stress by increasing the antioxidant capacity of cells, reducing inflammatory stimuli by inhibiting inflammatory factors, and delaying fibrosis by inhibiting the conversion of renal tubular epithelial cells into myofibroblasts^[5,6,7,8]^. Due to the complex pathogenesis of chronic kidney disease, the interaction between the various mechanisms is not yet clear, and there are insufficient reports on the clinical application of Quercetin, there are still some differences between clinical experiments and animal experiments, and there are many intrinsic and extrinsic environmental interference factors, we expect to further apply SQJZJN and Quercetin in clinical treatment, and further study the preventive and curative effects of SQJZJN and Quercetin on clinical chronic kidney disease^[9]^.

To fully evaluate the curative effects and mechanisms of Quercetin in treating CRF scientifically, the present study established a CRF rat model and intragastrically administered Quercetin to determine its effects. We constructed a regulatory network of SQJZJN-Quercetin-PIK3R1-CRF in a network pharmacological approach, investigated the target functions and molecular regulatory mechanisms to clarify the mechanisms of Quercetin on alleviating or delaying renal fibrosis^[10]^. The present study aimed to explore the mechanisms in the incidence and development of CRF, the clinical efficacy of SQJZJN and Quercetin, and the mechanisms of alleviating renal fibrosis.

## 2 Materials and methods

### 2.1 Animals

All animal experiments in the present study strictly followed the regulations of the Ethics Committee of Chongqing Traditional Chinese Medicine Hospital and the Declaration of Helsinki. A total of 160 male SPF SD rats weighing 200 ± 25 g were purchased from the Laboratory Animal Research Center of Army Medical University. All the SD rats were housed in cages, five in each under 60% air humidity, 20°C room temperature, and standardized lightening. Feedstuffs were supplied daily and water was given ad libitum.

### 2.2 Preparation of the CRF rat model

The 160 SD rats were fed for one week and 10 were randomly selected as the control group with normal diets. The remaining 150 rats were housed randomly in separate cages labeled with picric acid. The CRF rat model was established by intragastric administration of adenine solution^[11]^. The administered dose was prepared according to the body weight of SD rats. A suspension at 2.5% concentration was prepared with 100 mL normal saline supplemented with 2.5 g adenine, intragastrically administered at 200 mg·kg^−1^·d^−1^. Common feedstuffs and water were given ad libitum. The cycle of modeling lasted 28 days. After fasting for 4 h, blood samples were collected from the caudal vein to determine the levels of BUN, Cr, and UA.

### 2.3 Administration methods

The SQJZJN experiment used a normal control group, a CRF model group, and a treatment group with SQJZJN gavage, five rats in each group. The normal control group was fed with common feedstuffs; the CRF model group was given empty capsules by gavage during treatment; the SQJZJN treatment group was administered one SQJZJN capsule per day by intragastric gavage 8 weeks.

The animals of the Quercetin experiment were divided into five groups with five rats in each group; a normal control group, a model group, a low dose Quercetin group, a medium dose Quercetin group, and a high dose Quercetin group. The CRF rats were intragastrically administered Quercetin daily at doses of 0.75, 1.5, and 3 g/(kg·d) for 8 weeks according to corresponding body weight^[12]^.

### 2.4 Collection of renal tissue specimens

Before sample collection when the experiment was completed, the rats were anesthetized by intraperitoneally injected with 10% chloral hydrate. When the anesthesia was in effect, the chest and abdominal cavities were cut open to fully expose the heart. Blood samples were subsequently collected from the apex of the heart. The kidneys were taken out, washed with normal saline, and removed blood and fat non-renal tissues to observe its morphology^[13]^. The right kidney was placed in paraformaldehyde solution immediately for subsequent detection by optical microscopy and immunohistochemistry. The left kidney tissue was cut into pieces with scissors, placed in a cold preservation tube, and stored in a refrigerator at −80°C for RT-PCR and western blot assays.

### 2.5 HE staining

The detection was performed according to the instructions of the HE staining kit produced by Leagene Biotechnology Co., Ltd. The paraffin sections of kidney tissues were HE stained followed by image collection using a Mshot MF53 microscope from Guangzhou Mingmei Optoelectronic Technology Co., Ltd. All tissues of each section were observed at low magnification, including the general area and the specific morphology, and photographed the areas selected at 200 folds^[14]^.

### 2.6 Detection of cell apoptosis by flow cytometry

Cells were harvested, washed once with incubation buffer, and centrifugated at 500 r/min for 5 min. The cells were subsequently resuspended in 100 μL label solution, incubated for 10 min at room temperature in the dark, centrifugated at 500 r/min for 5 min, precipitated, and washed once with the incubation buffer. Fluorescence (SA-FLOUS) solution was added for incubation 20 min in the dark. The excitation light wavelength of flow cytometry was 488 nm. FITC fluorescence was detected by a 515 nm filter, and PI was detected at a wavelength over 560 nm^[15]^.

### 2.7 PIK3RI lentivirus packaging and tail vein injection of virus

The PIK3RI interfering lentivirus was packaged by Chongqing Biomedicine Biotechnology Co., Ltd. The brief procedures were as follows: The 293 cells were initially incubated in an oven at 37°C containing 5% CO_2_ for 8–24 h. Subsequently, transfection was carried out when the area of cell adherence reached more than 50% of the petri dish. A mixture of 400 μL core plasmid and packaging plasmid was dripped to a petri dish, mixed well by gently rocking the dish, placed in an oven, and incubated at 37°C containing 5% CO_2_. Following 12 h, the culture medium was sucked out, and an appropriate quantity of complete medium preheated at 37°C was supplemented and repeated incubation in the oven. The supernatant containing lentivirus was collected following 24 and 48 h transfection, respectively, passed through a 0.45 μm mesh, subpackaged, and stored at −80°C. The rats were fixed and injected with the virus at a dose of 1 × 10^7^ by tail vein, once every other day.

### 2.8 Co-precipitation assay of Quercetin and protein

The uniformly labeled 13 C Quercetin (>98.5% C atom labeled) was obtained from Isolife (NL). The Quercetin labeled with biotin and magnetic beads labeled with avidin were incubated at room temperature for 1 h. The magnetic beads were isolated using a magnetic rack, washed, and mixed with the total protein, and incubated using a vertical mixer overnight at 4°C. Following brief centrifugation, the magnetic beads were isolated using the magnetic rack and the supernatant was discarded. The magnetic beads were washed and supplemented with eluent and the proteins bound to biotin-labeled Quercetin were obtained for further analysis by western blot^[16]^.

### 2.9 Protein expression level was detected by western blot

Following the total protein extraction from the kidney tissues, the proteins were isolated using SDS-PAGE gel, and the antibodies against NLRP3, caspase1, p-PI3k, p-Akt, AQP1, AQP2, and β-actin were incubated respectively, and the corresponding secondary antibodies were subsequently incubated^[17]^. The protein bands were detected by ECL luminescence ultimately. The antibodies were listed in the following: NLRP3 (dilution 1:500, IGEE, China), caspase1 (dilution 1:500, IGEE, China), p-PI3k (dilution 1:500, IGEE, China), p-Akt (dilution 1:500, IGEE, China), AQP1 (dilution 1:1000, IGEE, China), AQP2 (dilution 1:1000, IGEE, China), β-actin (dilution 1:2000, IGEE, China), and HRP Goat anti-Rabbit IgG (dilution 1:2000, IGEE, China).

### 2.10 Immunofluorescence detection

Paraffin sections were cut at 4 μm thickness, baked at 60°C for 2 h, dewaxed, and hydrated with xylene and alcohol. The sections were placed in citrate buffer (pH = 6.0) for antigen repair, heated in an oven for 20 min and cooled down naturally to room temperature, rinsed with PBS 3 times, 5 min each time, blocked with 5% BSA for 30 min, incubated with primary antibodies (1:100) overnight at 4°C, rewarmed 30 min, and rinsed 3 times with PBS, 5 min each. Corresponding secondary antibodies (1:100) were added and incubated at room temperature for 1 h, rinsed 3 times with PBS, 5 min each time. DAPI incubation was performed in the dark for 5–10 min, following three cycles of washing with PBS, 1 min each cycle. The sections were sealed and placed under a microscope for photographing^[18]^.

### 2.11 Statistical analysis

The experimental data were statistically analyzed using SPSS 20.0 software. Measurement data were expressed as mean ± standard deviation (x ± s), and paired T-tests were used before and after treatment in the same group. Analysis of variance was used for comparison between groups. The value of P < 0.05 was considered the difference was significant, and P < 0.01 was considered the difference was extremely significant.

## 3. Results

We have innitially prepared the CRF rat model, the SQJZJN experiment and Quercetin experiment was performed to investigate its implication in treating chronic kidney failure rat. Then, the renal tissue was collected, the morphohistological changes were examined. Immunofluorescence detection was performed to explore the regulation of Quercetin on CRF-related genes and proteins. Ultimately, we found that SQJZJN and Quercetin can effectively alleviate CRF by inhibiting the PI3k/Akt pathwaythrough interfering PIK3R1.

### 3.1 SQJZJN can effectively treat CRF

**Figure 1.**
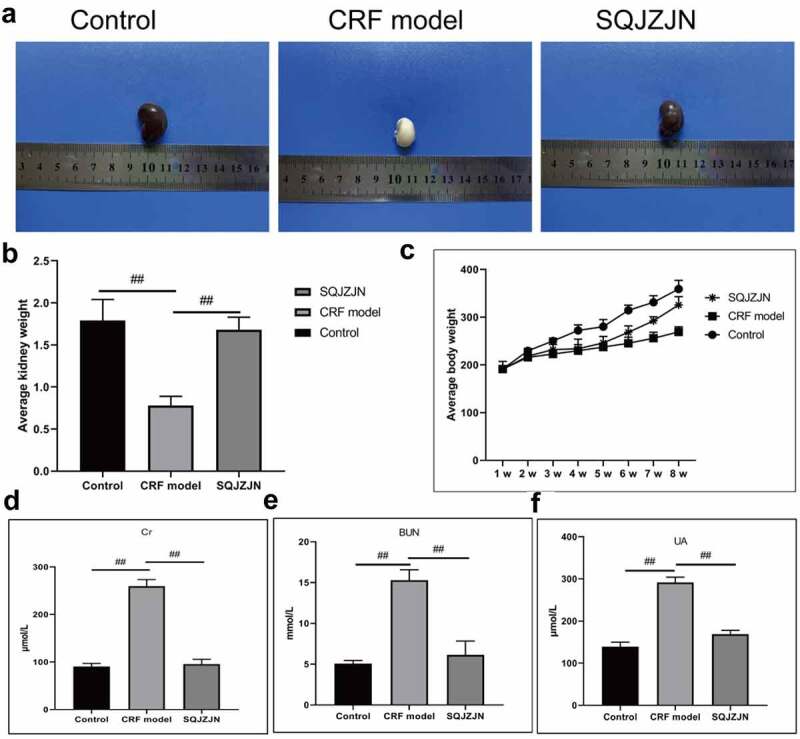
SQJZJN effectively improved CRF symptoms. A-B, Appearance and average weight of rat kidneys in the normal control, model, and SQJZJN treatment groups from left to right. C, Weight of the rats. D, Content of Cr. E, Content of BUN. F, Content of UA. ##, p < 0.01

The CRF model rats were intragastrically administered SQJZJN daily. The normal kidneys were bean-like reddish brown with a hard texture. And the size was normal, with gloss and regular morphology and no swelling. After dissection, a clear boundary was found between the skin and medulla. The cortex was reddish brown, and the medulla was light red. The renal capsule was tightly integrated and could not be separated easily. The kidney size in the other groups was enlarged differently. The model group, in particular, was the most obvious which was twice as big as the normal rats. Meanwhile, the kidney of this group was pale in color with uneven surfaces, turned into a ‘big white kidney’. The size of the kidney in the SQJZJN treatment group was between the normal and model groups, showing dark red and a few white particles on the surface. The renal capsule adhered to tissues, and the boundary between the cortex and the medulla was visible (Figure 1a). The kidney weight in the SQJZJN treatment group was between the normal and model groups, almost equivalent to normal group, showing good treatment in kidney, while the kidney in model group was measured lost nearly double the weight (Figure 1b).

**Figure 2.**
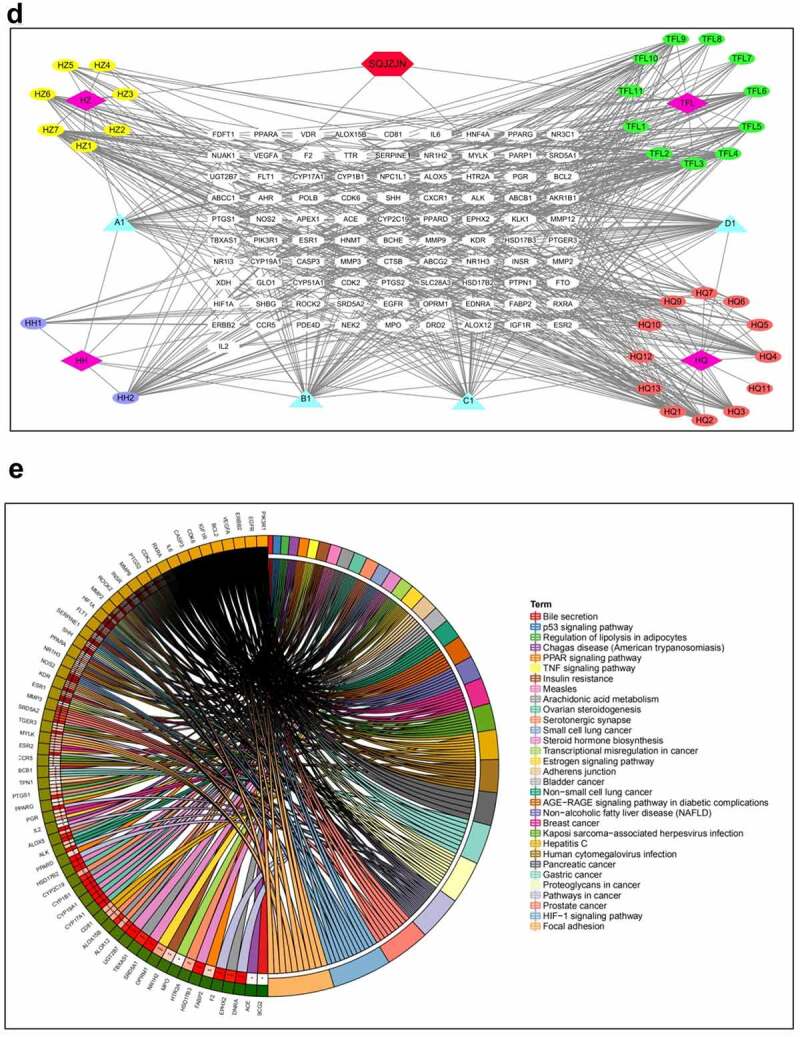
Continued

Eight weeks later, the weight of rats in the model group was markedly lower than in the control group, while that in the SQJZJN group was lower than in the control group but much higher than the model group (Figure 1c). Moreover, compared with the normal group, the levels of Cr, BUN, and UA in the model group were markedly increased (P < 0.01). Compared with the model group, the levels of Cr, BUN, and UA decreased dramatically by SQJZJN administration, indicating that SQJZJN could effectively alleviate and treat CRF (Figure 1d-1f).**Figure 2.**
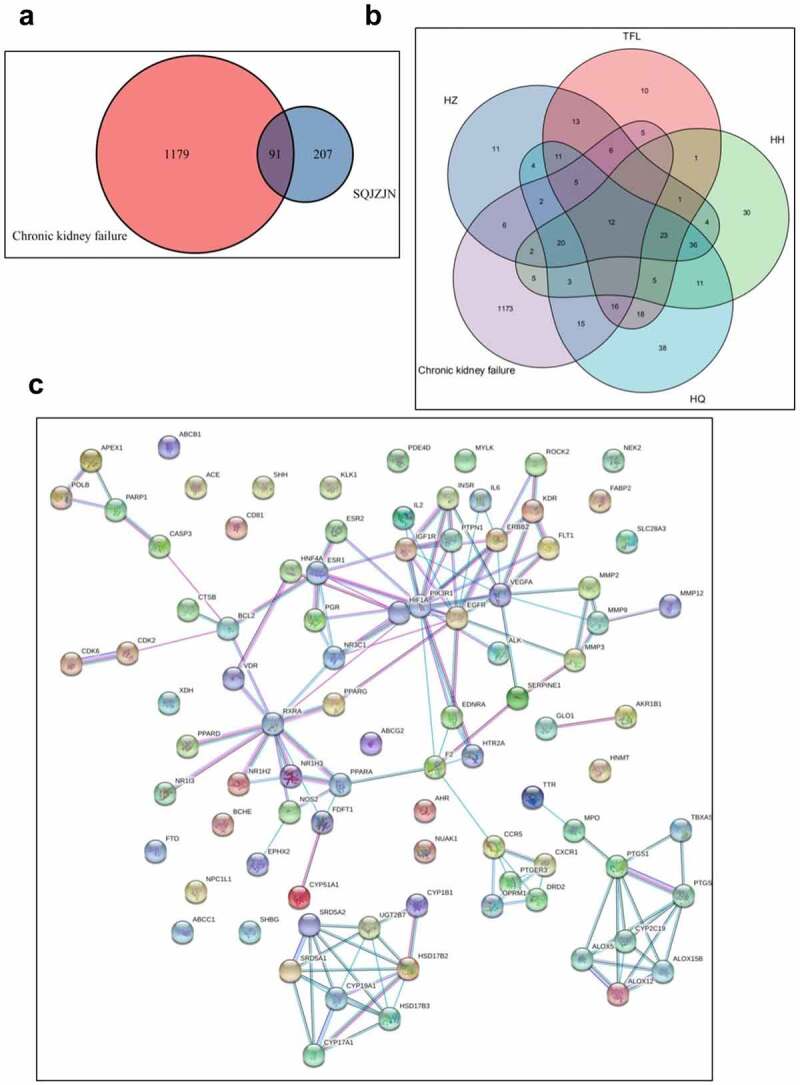
Network pharmacological analysis of SQJZJN-ingredients-targets-disease. A, Overlapped genes of the corresponding component targets of SQJZJN and CRF associated target genes. B, Overlapped genes between Polygonum cuspidatum Siebold & Zucc (Huzhang), Smilax glabra Roxb. (Tufuling), Sophora japonica L. (Huaihua), and Astragalus membranaceus (Fisch.) Bunge (Huangqi) and the disease targets. C, PPI network diagram. D, Network diagram of drug-active ingredients-target genes. E, Target gene enrichment chart

### 3.2 Network pharmacological analysis of SQJZJN

To clarify the mechanisms of SQJZJN, we undertook a network pharmacological approach to this prescription^[19]^. The main components of SQJZJN including Polygonum cuspidatum Siebold & Zucc (Huzhang), Smilax glabra Roxb. (Tufuling), Sophora japonica L. (Huaihua), and Astragalus membranaceus (Fisch.) Bunge (Huangqi) were sorted out from the TCMSP database. The compounds with bioavailability (OB) ≥ 30% and drug likeness (DL) ≥ 0.18 were selected as the main active ingredients of the prescription. The statistical results were illustrated in Supplement Table 1. The corresponding ingredient targets of SQJZJN were intersected with the CRF disease targets downloaded from the Genecard database. A total of 1,270 CRF disease targets and 298 targets of SQJZJN were sorted out. After analysis, 91 overlapped genes were obtained (Supplement Table 2). A venn diagram was plotted as Figure 2a. Twelve overlapped genes were found between the action targets of Polygonum cuspidatum Siebold & Zucc (Huzhang), Smilax glabra Roxb. (Tufuling), Sophora japonica L. (Huaihua), and Astragalus membranaceus (Fisch.) Bunge (Huangqi), and the disease targets (Supplement Table 3), and its venn diagram was shown in Figure 2b. The protein-protein interaction (PPI) of the 91 genes was analyzed and the results were illustrated in Figure 2c. As PIK3R1 located in the center with the most edges, it could be selected either as a candidate gene or a signaling pathway for further research. Protein interaction network analysis was a PPI diagram constructed based on the existing research results or predicted results. It provided evidence for subsequent Co-IP experiments and revealed the direct regulatory effects of proteins. The network diagram was plotted with the overlapped genes, the drug SQJZJN, and its active ingredients. A total of 36 main active ingredients from the four main components Polygonum cuspidatum Siebold & Zucc (Huzhang), Smilax glabra Roxb. (Tufuling), Sophora japonica L. (Huaihua), and Astragalus membranaceus (Fisch.) Bunge (Huangqi) in SQJZJN had overlapped genes with the disease targets. The drug-active ingredient-target gene network was plotted as Figure 2d. To clarify the functions of SQJZJN targets and the role of potential targets in the signaling pathways, GO analysis and KEGG pathway analysis of the 91 targets (Figure 2e) were performed using Cytoscape ClueGO software and the enrichment results were under visual processing. The analysis results demonstrated that Polygonum cuspidatum Siebold & Zucc (Huzhang), Smilax glabra Roxb. (Tufuling), Sophora japonica L. (Huaihua), and Astragalus membranaceus (Fisch.) Bunge (Huangqi) all contained Quercetin, and Quercetin might interact with multiple target genes. We therefore speculated that Quercetin might be used as the main functional ingredient of SQJZJN to treat CRF.

### 3.3 Quercetin can effectively alleviate CRF

**Figure 3.**
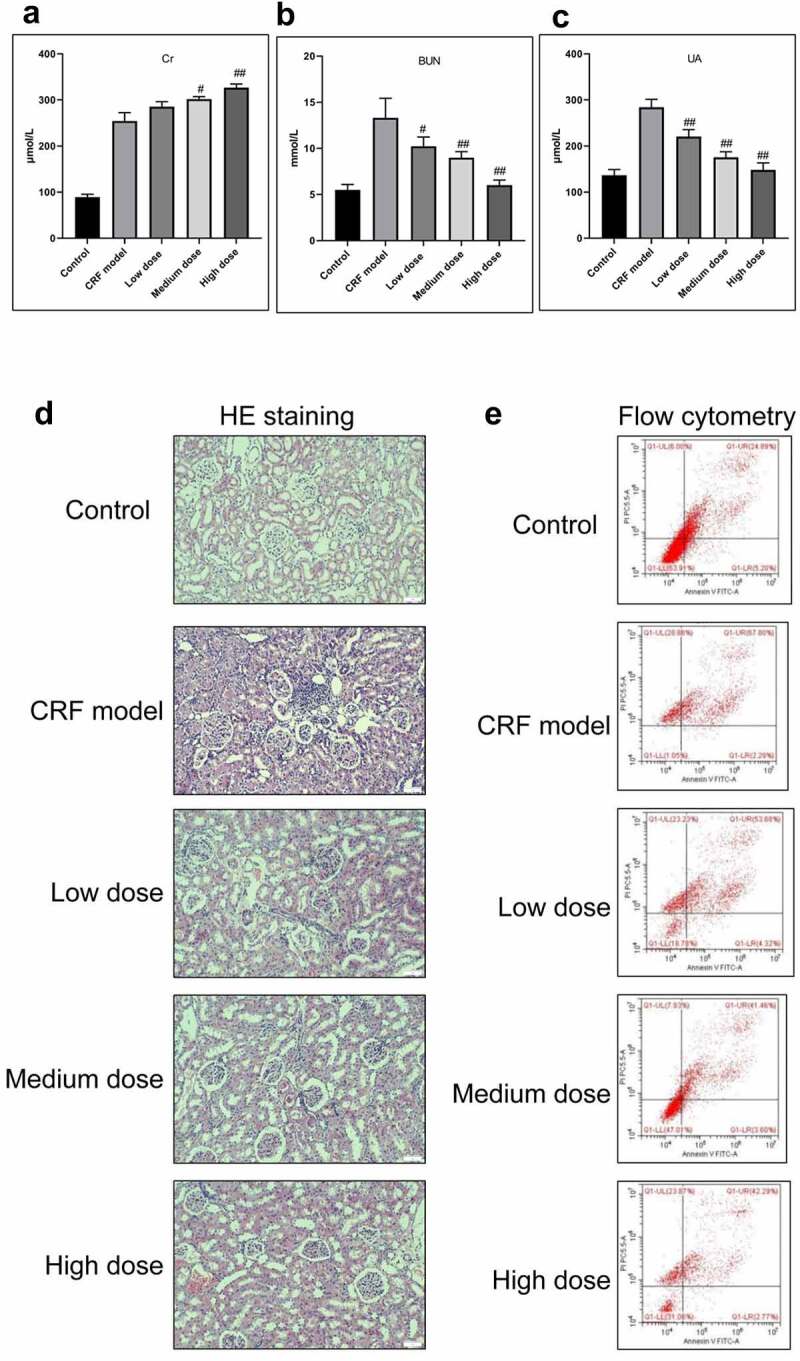
Effective treatment of Quercetin to CRF. The rats by intragastric administration of Quercetin were divided into a normal control group, a model group, a low dose Quercetin group, a medium dose Quercetin group, and a high dose Quercetin group (n = 5). The CRF rats were intragastrically administered Quercetin daily at doses of 0.75, 1.5, and 3 g/(kg·d) according to the body weight for 8 weeks. A, Content of Cr. B, Content of BUN. C, Content of UA. D, HE staining. E, Detection of cell apoptosis by flow cytometry. #, p < 0.05. ##, p < 0.01

The CRF rats were intragastrically administered with low, medium, and high doses of Quercetin. Compared with the model group, the levels of Cr, BUN, and UA were dramatically decreased following the administration of medium and high doses of Quercetin (Figure 3a-3c).

**Figure 4.**
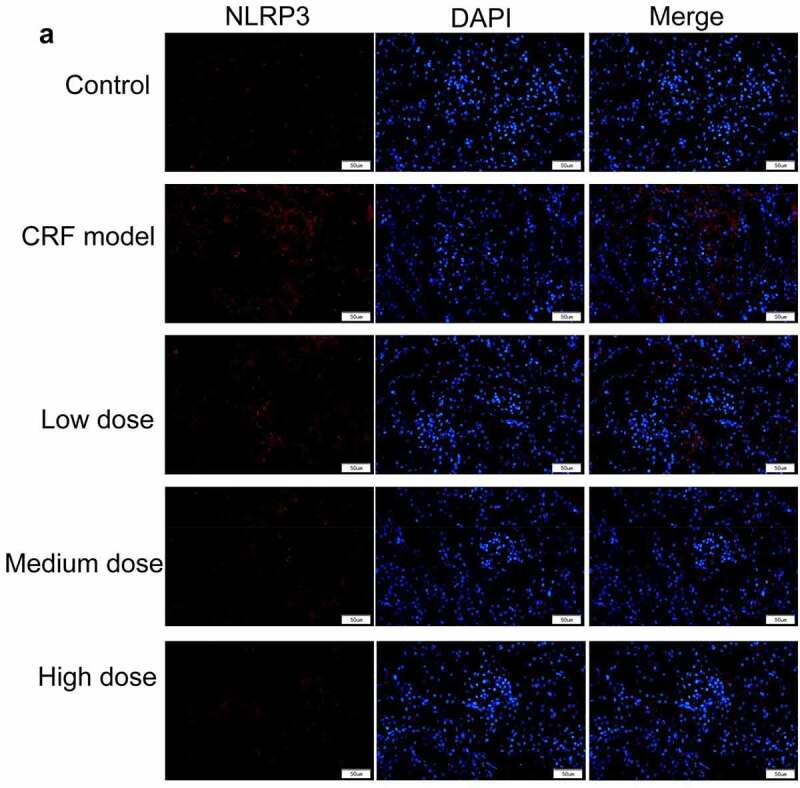
Regulatory effects of Quercetin on CRF genes and proteins. A-D, Expression of NLRP3, caspase1, AQP1, and AQP2 was detected by immunofluorescence. E, Statistical results of the average fluorescence intensity. F-G, Expression of p-PI3K, p-AKT, NLRP3, Caspase1, AQP1, and AQP2 by western blot. H, Grayscale statistical results of western blot. #, p < 0.05. ##, p < 0.01. Scale bar: 50 μm

**Figure 4.**
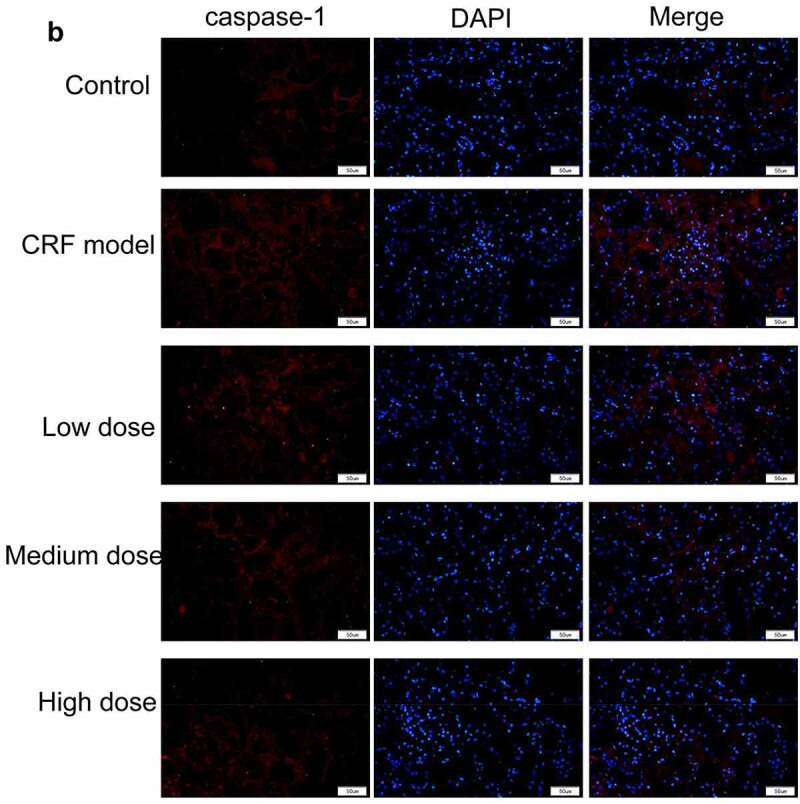
Continued

**Figure 4.**
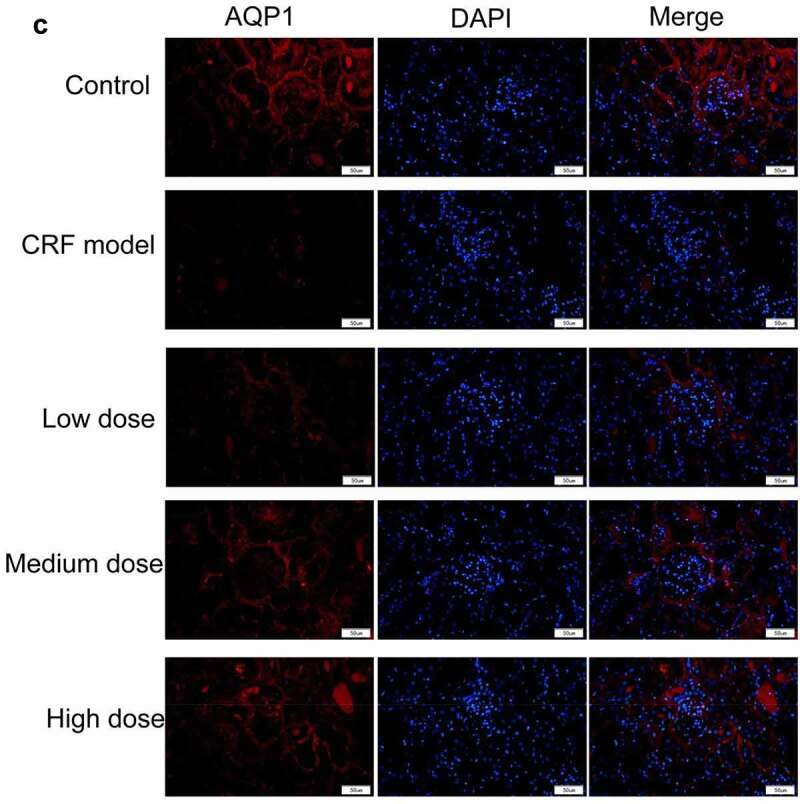
Continued

**Figure 4.**
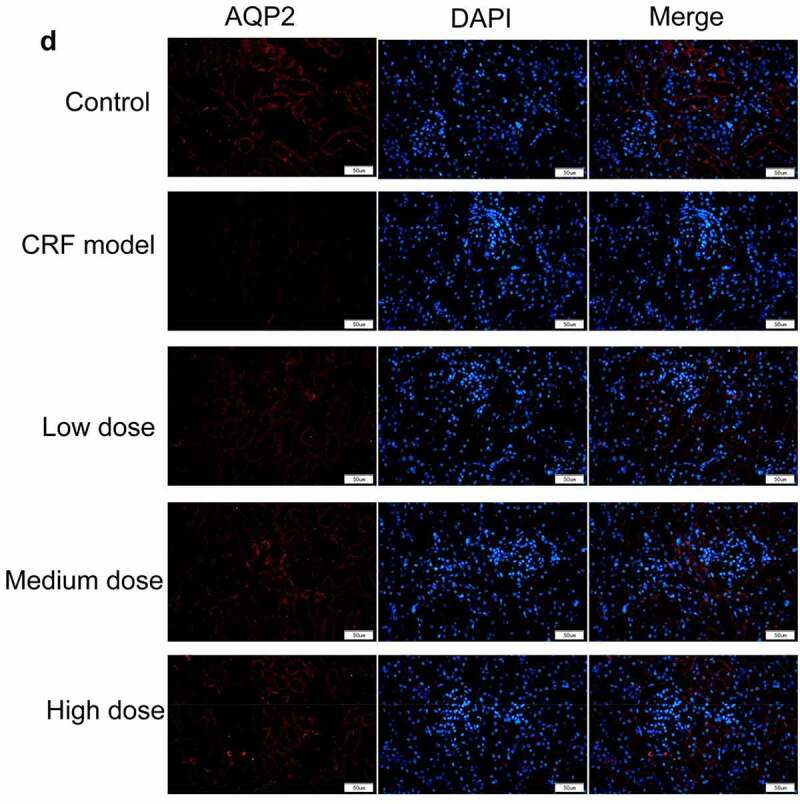
Continued

**Figure 4.**
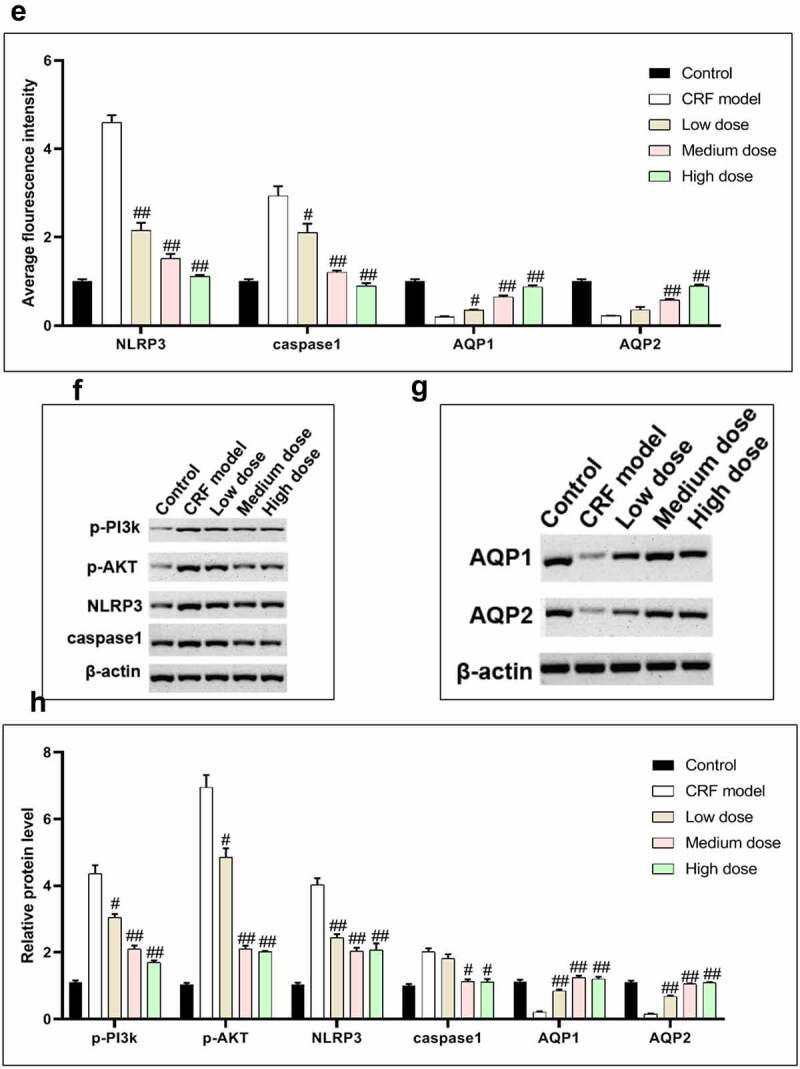
Continued

The glomerular structure was normal in the normal group. In the model group, however, the glomerular developed segmental or spherical sclerosis, balloon wall adherence, and partially thickened basement membrane. Additionally, the glomerular mesangium displayed focal and diffusive enlargement but the glomerular mesangial cells and mesangial matrix increased. The capillaries were narrowed and the kidney capsule was thickened by compression^[20,21]^. In the Quercetin treatment group, the glomerular capillary cavity was partially opened, partial glomerular mesangium was slightly enlarged with a minor visible increase of the mesangial matrix (Figure 3d).

We subsequently determined the effect of Quercetin on cell apoptosis. The results of flow cytometry indicated a large number of cell apoptosis in the model group. Apoptosis in the model group was markedly higher than in the normal group whereas apoptosis in the Quercetin treatment group was dramatically reduced compared with the model group and the highest decrease was in the high dose Quercetin group (Figure 3e), suggesting that Quercetin could effectively minimize cell apoptosis.

### 3.4 Regulation of Quercetin on CRF-related genes and proteins

The expression levels of NLRP3, caspase1, AQP1, and AQP2 in each group were detected by immunofluorescence. The results revealed that the mean fluorescence intensity ratio of NLRP3 and caspase1 in the model kidney tissues was markedly higher than in the normal group. Compared with the model group, the mean fluorescence intensity ratio was dramatically decreased in the Quercetin treatment group, representing the most dramatic decrease. The mean fluorescence intensity ratio of AQP1 and AQP2 in the model group was markedly lower than that in the normal group, whereas that of AQP1 and AQP2 increased in the Quercetin group (Figure 4a-4e). Western blot results showed consistent trends with the immunofluorescence results (figure 4f-h).

### 3.5 Mechanisms of Quercetin on CRF

**Figure 5.**
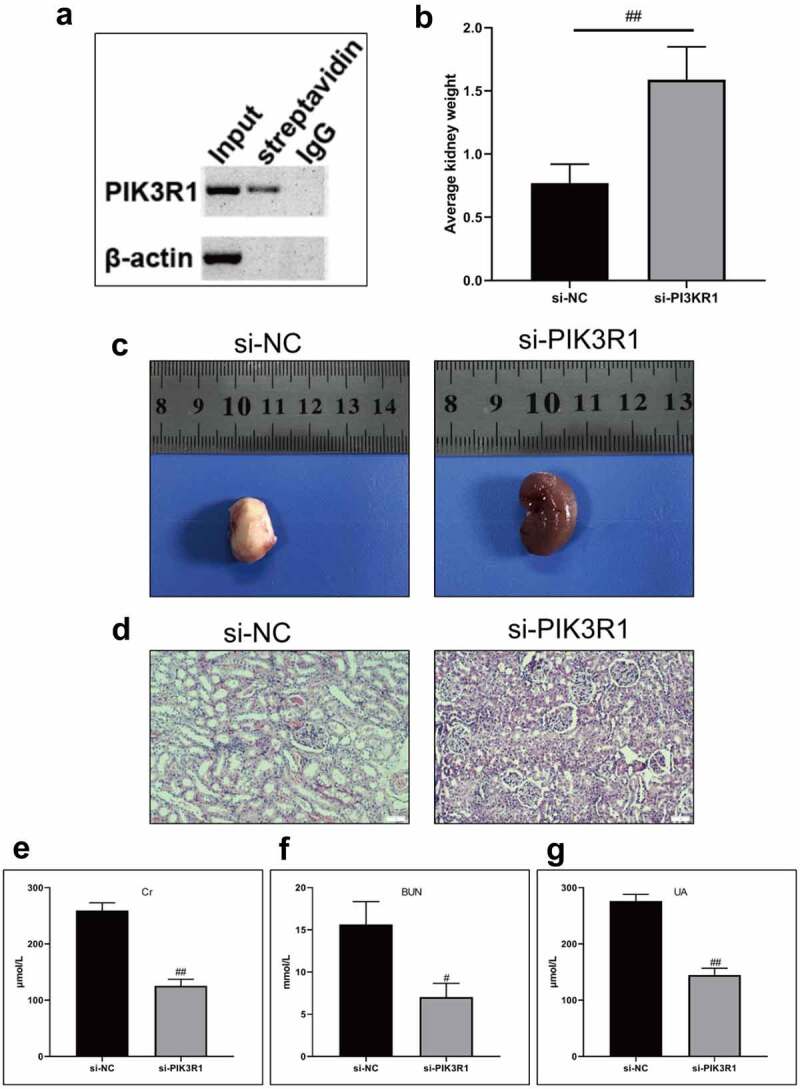
Quercetin treats CRF by regulating PIK3R1. A, PIK3R1 results by western blot after biotin-labeled Quercetin incubated with total kidney protein. B-C, Average weight and appearance of rat kidney after interfering PIK3R1. From left to right, the model group+si-NC, the model group+si-PIK3R1 group. D, HE staining. E, Content of Cr. F, Content of BUN. G, Content of UA. #, p < 0.05. ##, p < 0.01

After being labeled with biotin, Quercetin was incubated with the total protein of the kidney tissues, the protein conjugated with biotin-labeled Quercetin was extracted using affinity streptomycin. The results revealed that Quercetin could bind to PIK3R1 detected by western blot assay (Figure 5a). We speculated that Quercetin could bind to PIK3R1 and inhibited its activity, thereby mediating the PI3K/AKT pathway. We subsequently coated the PIK3R1 interfering lentivirus and injected it into the CRF model rats by tail vein. Eight weeks later, the kidney weight and appearance was improved in the si-PIK3R1 group (Figure 5b-5d), whose phenotype was superior to that of the model group. Additionally, the levels of Cr, BUN, and UA were markedly reduced in the si-PIK3R1 group (Figure 5e-5g).

**Figure 6.**
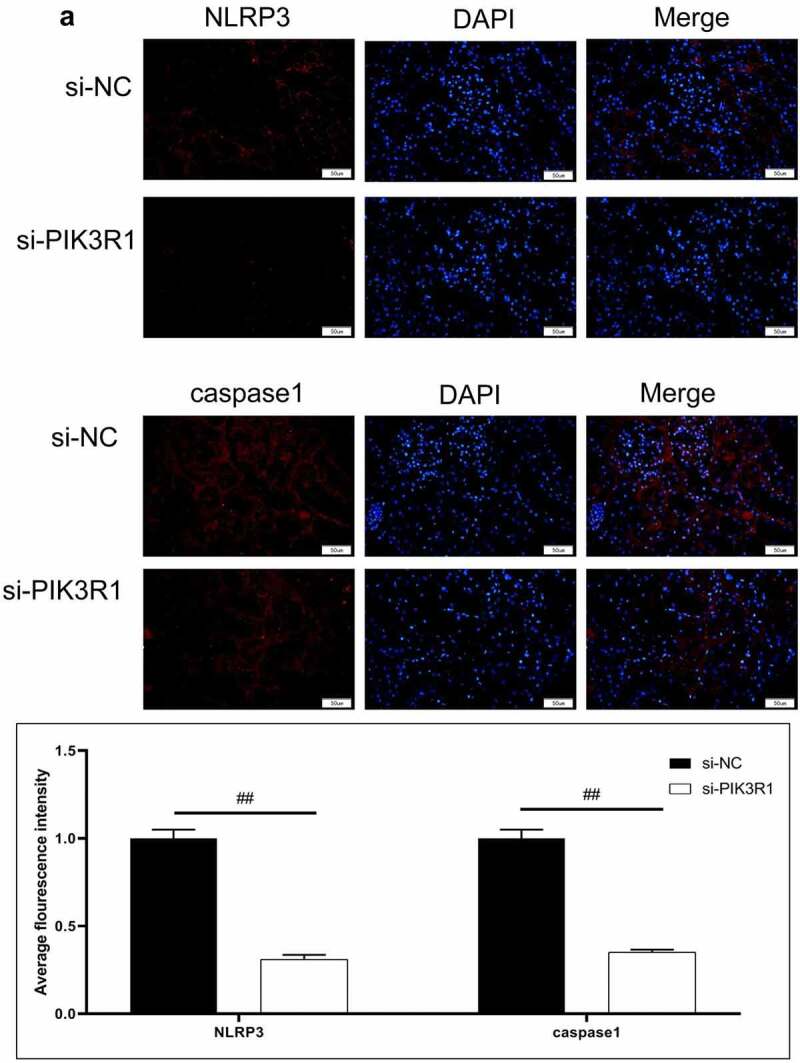
Regulatory effects on CRF genes and proteins by interfering PIK3R1. A, Expression of NLRP3 and caspase1 was detected by immunofluorescence. B, Expression of AQP1 and AQP2 was detected by immunofluorescence. C-D, Expression of p-PI3K, p-AKT, NLRP3, caspase1, AQP1, and AQP2 was detected by western blot. E, Grayscale statistical results by western blot. #, p < 0.05. ##, p < 0.01. Scale bar: 50 μm

**Figure 6.**
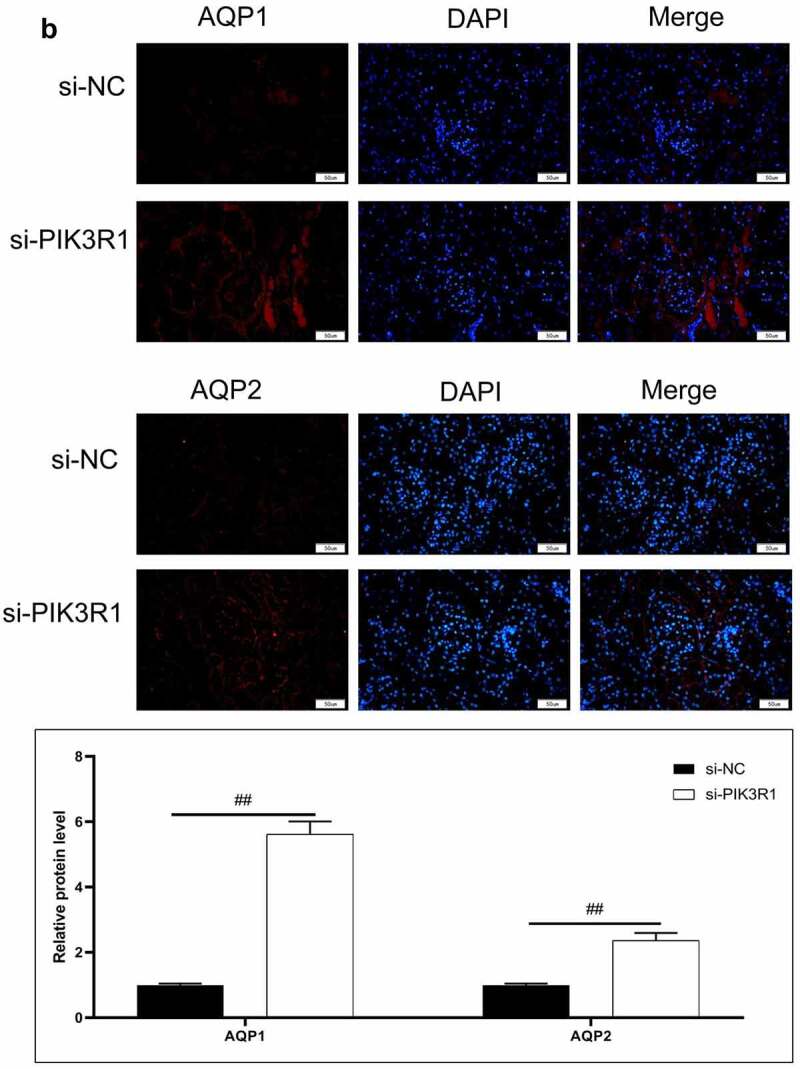
Continued

**Figure 6.**
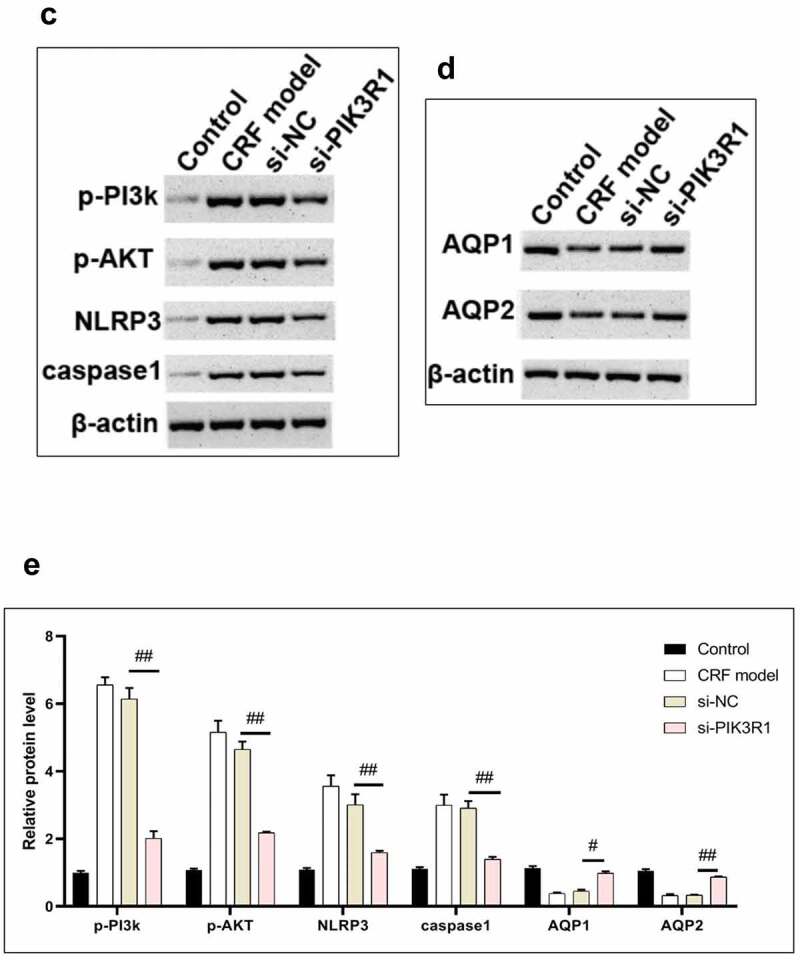
Continued

The expression levels of NLRP3, caspase1, AQP1, and AQP2 in each group were detected by immunofluorescence. The results showed that the mean fluorescence intensity ratio of NLRP3 and caspase1 in the si-PIK3R1 group was dramatically lower than in the si-NC group (Figure 6a). The mean fluorescence intensity of AQP1 and AQP2 was higher than that of the si-NC group (Figure 6b). The trend of western blot results was consistent with those of immunofluorescence (Figure 6c-6e). The previously described experiments indicated that renal apoptosis was decreased and the PI3k/Akt pathway was inhibited by interfering PIK3R1.

Taken together, Quercetin could bind to PIK3R1 and inhibit the PI3k/Akt pathway, thereby minimizing renal fibrosis and apoptosis in CRF rats. By regulating AQP1 and AQP2, both the retention of water and the accumulation of toxins were reduced.

## 4 Discussion

CRF is the consequence of CKD development. Its clinical manifestations are characterized by the retention of toxic metabolites, disorders of water, electrolyte, and acid-base balance, accompanying multiple organ impairment throughout the body^[22]^. As CKD mortality has increased by 14.7% in China in the past 15 years, this disease has emerged into a problem to be solved immediately for the effective prevention of CRF^[23]^. Despite there is no specific treatment, TCM has exerted notable effects on the prevention and treatment of chronic diseases^[23]^. Glomerular filtration rate (GFR) is a valuable indicator to evaluate the kidney filtration function. To estimate GFR clinically, a series of equations and relevant simplified equations have been developed by the US Modification of Diet in Renal Diseases Study (MDRD) based on gender, race, age, and levels of serum creatinine (Scr)^[24]^. The chronic kidney disease epidemiology collaboration (CKD-EPI) equation is a newly developed GFR estimation equation by the US CKD-EPI, and its predictive performance was superior to the simplified MDRD equation reported by studies on CKD patients and healthy people in the United States^[25,26]^. Researchers in China have modified a simplified MDRD equation and improved its compatibility for Chinese users. Compared with the simplified MDRD equation, the estimation of CKD-EPI is more accurate^[27]^, which was applied for the GFR estimation in the present study. The levels of Cr, BUN, and UA in the SQJZJN group, the Quercetin group, and the interfering PIK3R1 group were lower than in the model group.

Modern medical research has demonstrated that the primary pathological changes of CRF include general atrophy and fibrosis of glomerulus, hypertrophy, necrosis, and occlusion of renal tubular, leading to glomerular sclerosis and interstitial fibrosis^[28]^. Mesangial cells act as the supporting structure of glomerular capillaries, their contractions are essential to maintain normal renal physiological functions and the development of renal lesions. Once the kidney is impaired, glomerular mesangial cells proliferate, and ECM accumulation represents a characteristic change of glomerulosclerosis. Consequently, the inhibition of abnormal growth of mesangial cells can delay the progression of CRF pathology. This experimental study verified the pathological changes in CRF treated by SQJZJN and Quercetin to a certain extent. The anatomic appearance of the kidneys in the SQJZJN group and the Quercetin treatment group was markedly improved, and the color of the ‘big white kidney’ in the CRF model group turned into red and white. The pathological sections by HE staining revealed that the ratio of the glomerular mesangium/glomerular area was dramatically reduced both in the SQJZJN group and Quercetin treatment group compared with the model group. The present study suggested that SQJZJN and Quercetin inhibited the proliferation of glomerular mesangium, reduced the degree of glomerular sclerosis, and delayed the progression of CRF. And compared with it, Hu Yang et al found that quercetin could reduce the abnormal histopathological renal changes in chronic kidney model, including the chronic interstital inflammation^[^^8^^]^.

PI3K, a neural signal transduction enzyme, can be activated by tyrosine kinase receptor, G protein-coupled receptor/cytokine receptor, and Ras protein-related GDP enzyme receptor, so that it can promote the proliferation, survival, adhesion, and differentiation of cells participating in the cytoskeletal organization^[29,30]^. Akt serves as a downstream serine/threonine kinase of PI3k and involves in cell proliferation and differentiation by activating downstream factors HIF-1a and mTOR^[31,32]^. PI3K/AKT works as an important signal regulatory mechanism for human cell growth and development^[33,34,35]^. It mediates diverse cellular processes including cell growth, development, division, differentiation, apoptosis, and even intercellular functions. During renal fibrosis, the PI3k/Akt pathway links to inflammatory impairment, parenchymal cell apoptosis, epithelial cell transdifferentiation, and the proliferation of mesenchymal cells namely fibroblasts. The findings of HE staining in this experiment indicated that Quercetin produced a certain effect on the alleviation of renal fibrosis, suggesting that Quercetin exerted a notable inhibitory effect on the PI3k/Akt pathway. The expression of p-PI3k and p-Akt in the model kidney tissues was markedly higher than in the normal group (P < 0.01). Compared with the model group, the expression of p-PI3k and p-Akt in the Quercetin treatment group was dramatically reduced (P < 0.01). Quercetin was hypothesized that it was capable of inhibiting the PI3k/Akt pathway by binding to PIK3R1 and mediating the phosphorylation of PI3k and Akt and this is consistent with previous study^[36]^.

AQP1 locates on the proximal tubules, the lateral lumen of renal tubule epithelial cells, and basolateral cell membranes^[37]^. It also participates in the reabsorption of water molecules in the tubule fluid. The expression of AQP1 can also be regulated by hypertonicity and angiotensin II. AQP1 knockout mice develop polyuria and reduced urine osmotic pressure^[38]^. AQP2 is distributed in the connective ducts and manifold cells, mostly on the lateral lumen cell membranes and the intracellular vesicle membranes. Two regulatory mechanisms of the water transport molecule AQP2 contain short-term regulation and long-term regulation. The former predominantly mediates the transporting processes of exocytosis and endocytosis of the synthesized AQP2 in intracellular vesicles and membranes, which usually takes within 5–30 min. The latter takes 24 h or a longer period, mainly regulating the transcriptional expression of AQP2^[39,40]^. This experimental study demonstrated that Quercetin could act on the CRF rat kidneys, decompose food and excrete water by regulating AQP1 and AQP2, thereby reducing both the retention of water and the accumulation of toxins.

## 5 Conclusion

We have labeled the Quercetin with biotin and incubated with the total protein of the kidney tissues, the protein conjugated with biotin-labeled Quercetin was extracted using affinity streptomycin. The western blot assay showed that the protein conjugated with biotin-labeled Quercetin is capable of binding to PI3KR1. Thus, the Quercetin might combine with PIK3R1 and minimize renal fibrosis and apoptosis in CRF rats by inhibiting the PI3k/Akt pathway. By regulating AQP1 and AQP2, both the retention of water and the accumulation of toxins were reduced. The chronic kidney failure could alleviated by Quercetin through targeting PI3KR1, and further appears to be a promising candidate with chemotherapeutic potential and warrants further research.

## Supplementary Material

Supplemental Material

## Highlights:

Quercetin minimized renal fibrosis and apoptosis in CRF rats.

Quercetin could bind to PIK3R1.

Quercetin could increase the expression of AQP1 and AQP2.
