# Supplementary material for: Quercetin alleviates chronic renal failure by targeting the PI3k/Akt pathway
Source: Bioengineered. 2021 Sep 16;12(1):6538–58. doi: 10.1080/21655979.2021.1973877 (PMC8806539; doi:10.1080/21655979.2021.1973877)
Supplement: Supplemental Material [file KBIE_A_1973877_SM7825.docx]

Supplement Table 1 Main active components of SQJZJN

|  |  | [Mol ID](https://tcmspw.com/tcmspsearch.php?qr=Polygoni%20Cuspidati%20Rhizoma%20Et%20Radix&qsr=herb_en_name&token=fb630e8dc0e869fac800bccd076cc9f4) | [Molecule Name](https://tcmspw.com/tcmspsearch.php?qr=Polygoni%20Cuspidati%20Rhizoma%20Et%20Radix&qsr=herb_en_name&token=fb630e8dc0e869fac800bccd076cc9f4) |
| --- | --- | --- | --- |
| Polygonum Cuspidatum (Huzhang) | HZ1 | MOL013281 | [6,8-Dihydroxy-7-methoxyxanthone](https://tcmspw.com/molecule.php?qn=13281) |
|  | HZ2 | MOL013287 | [Physovenine](https://tcmspw.com/molecule.php?qn=13287) |
|  | HZ3 | MOL013288 | [Picralinal](https://tcmspw.com/molecule.php?qn=13288) |
|  | HZ4 | MOL002259 | [Physciondiglucoside](https://tcmspw.com/molecule.php?qn=2259) |
|  | HZ5 | MOL002268 | [rhein](https://tcmspw.com/molecule.php?qn=2268) |
|  | HZ6 | MOL000492 | [(+)-catechin](https://tcmspw.com/molecule.php?qn=492) |
|  | HZ7 | MOL000006 | [luteolin](https://tcmspw.com/molecule.php?qn=6) |
|  | A1 | MOL000358 | [beta-sitosterol](https://tcmspw.com/molecule.php?qn=358) |
|  | B1 | MOL000098 | [Quercetin](https://tcmspw.com/molecule.php?qn=98) |
| Rhizoma Smilacis Glabrae (Tufuling) | TFL1 | MOL013118 | [Neoastilbin](https://tcmspw.com/molecule.php?qn=13118) |
|  | TFL2 | MOL013129 | [(2R,3R)-2-(3,5-dihydroxyphenyl)-3,5,7-trihydroxychroman-4-one](https://tcmspw.com/molecule.php?qn=13129) |
|  | TFL3 | MOL001736 | [(-)-taxifolin](https://tcmspw.com/molecule.php?qn=1736) |
|  | TFL4 | MOL000359 | [sitosterol](https://tcmspw.com/molecule.php?qn=359) |
|  | TFL5 | MOL004328 | [naringenin](https://tcmspw.com/molecule.php?qn=4328) |
|  | TFL6 | MOL000449 | [Stigmasterol](https://tcmspw.com/molecule.php?qn=449) |
|  | TFL7 | MOL004567 | [isoengelitin](https://tcmspw.com/molecule.php?qn=4567) |
|  | TFL8 | MOL004575 | [astilbin](https://tcmspw.com/molecule.php?qn=4575) |
|  | TFL9 | MOL004576 | [taxifolin](https://tcmspw.com/molecule.php?qn=4576) |
|  | TFL10 | MOL004580 | [cis-DihydroQuercetin](https://tcmspw.com/molecule.php?qn=4580) |
|  | TFL11 | MOL000546 | [diosgenin](https://tcmspw.com/molecule.php?qn=546) |
|  | A1 | MOL000358 | [beta-sitosterol](https://tcmspw.com/molecule.php?qn=358) |
|  | B1 | MOL000098 | [Quercetin](https://tcmspw.com/molecule.php?qn=98) |
| Flos Sophorae (Huaihua) | HH1 | MOL005935 | [N-[6-(9-acridinylamino)hexyl]benzamide](https://tcmspw.com/molecule.php?qn=5935) |
|  | HH2 | MOL005940 | [Quercetin-3'-methyl ether](https://tcmspw.com/molecule.php?qn=5940) |
|  | C1 | MOL000354 | [isorhamnetin](https://tcmspw.com/molecule.php?qn=354) |
|  | A1 | MOL000358 | [beta-sitosterol](https://tcmspw.com/molecule.php?qn=358) |
|  | D1 | MOL000422 | [kaempferol](https://tcmspw.com/molecule.php?qn=422) |
| Astragalus Membranaceus (Huangqi) | HQ1 | MOL000211 | [Mairin](https://tcmspw.com/molecule.php?qn=211) |
|  | HQ2 | MOL000239 | [Jaranol](https://tcmspw.com/molecule.php?qn=239) |
|  | HQ3 | MOL000296 | [hederagenin](https://tcmspw.com/molecule.php?qn=296) |
|  | HQ4 | MOL000033 | [(3S,8S,9S,10R,13R,14S,17R)-10,13-dimethyl-17-[(2R,5S)-5-propan-2-yloctan-2-yl]-2,3,4,7,8,9,11,12,14,15,16,17-dodecahydro-1H-cyclopenta[a]phenanthren-3-ol](https://tcmspw.com/molecule.php?qn=33) |
|  | HQ5 | MOL000371 | [3,9-di-O-methyNLRP3issolin](https://tcmspw.com/molecule.php?qn=371) |
|  | HQ6 | MOL000378 | [7-O-methylisomucronulatol](https://tcmspw.com/molecule.php?qn=378) |
|  | HQ7 | MOL000380 | [(6aR,11aR)-9,10-dimethoxy-6a,11a-dihydro-6H-benzofurano[3,2-c]chromen-3-ol](https://tcmspw.com/molecule.php?qn=380) |
|  | HQ8 | MOL000387 | [Bifendate](https://tcmspw.com/molecule.php?qn=387) |
|  | HQ9 | MOL000392 | [formononetin](https://tcmspw.com/molecule.php?qn=392) |
|  | HQ10 | MOL000417 | [Calycosin](https://tcmspw.com/molecule.php?qn=417) |
|  | HQ11 | MOL000433 | [FA](https://tcmspw.com/molecule.php?qn=433) |
|  | HQ12 | MOL000438 | [(3R)-3-(2-hydroxy-3,4-dimethoxyphenyl)chroman-7-ol](https://tcmspw.com/molecule.php?qn=438) |
|  | HQ13 | MOL000439 | [isomucronulatol-7,2'-di-O-glucosiole](https://tcmspw.com/molecule.php?qn=439) |
|  | HQ14 | MOL000442 | [1,7-Dihydroxy-3,9-dimethoxy pterocarpene](https://tcmspw.com/molecule.php?qn=442) |
|  | C1 | MOL000354 | [isorhamnetin](https://tcmspw.com/molecule.php?qn=354) |
|  | D1 | MOL000422 | [kaempferol](https://tcmspw.com/molecule.php?qn=422) |
|  | B1 | MOL000098 | [Quercetin](https://tcmspw.com/molecule.php?qn=98) |

Supplement Table 2 Overlapped genes of the corresponding component targets of SQJZJN and CRF associated target genes

| VEGFA | HIF1A | PTGS2 | XDH | FLT1 | CXCR1 | CYP2C19 | PDE4D | ALOX15B |
| --- | --- | --- | --- | --- | --- | --- | --- | --- |
| PPARG | HNF4A | SLC28A3 | CASP3 | CDK6 | INSR | NR3C1 | PGR | NR1H3 |
| F2 | MMP12 | SHBG | TTR | PARP1 | KDR | AHR | UGT2B7 | CDK2 |
| MMP9 | KLK1 | BCL2 | NUAK1 | CYP19A1 | ACE | EDNRA | NR1H2 | CYP17A1 |
| MPO | MMP3 | BCHE | EPHX2 | CYP1B1 | VDR | PPARD | NR1I3 | CYP51A1 |
| ESR1 | ABCC1 | PTGS1 | ERBB2 | IGF1R | IL6 | RXRA | CD81 | CTSB |
| ABCB1 | GLO1 | SRD5A1 | ESR2 | APEX1 | SERPINE1 | SHH | ROCK2 | HTR2A |
| EGFR | ALOX5 | OPRM1 | ALK | HSD17B2 | CCR5 | TBXAS1 | DRD2 | HNMT |
| MMP2 | FTO | PIK3R1 | ALOX12 | MYLK | IL2 | SRD5A2 | FDFT1 | HSD17B3 |
| ABCG2 | PTPN1 | POLB | AKR1B1 | NEK2 | PPARA | PTGER3 | FABP2 | NPC1L1 |
| NOS2 |  |  |  |  |  |  |  |  |

Supplement Table 3 Overlapped genes between Polygonum Cuspidatum (Huzhang), Rhizoma Smilacis Glabrae (Tufuling), Flos Sophorae (Huaihua) and Astragalus Membranaceus (Huangqi) and the disease targets

| MMP9 | ESR2 | ABCC1 |
| --- | --- | --- |
| ABCB1 | CYP19A1 | PTPN1 |
| MMP2 | CYP1B1 | BCHE |
| ABCG2 | KDR | SRD5A1 |
